# Choosing Castration: A Thematic Analysis of the Perceived Pros and Cons of Genital Injuries and Ablation by Men Who Voluntarily Sought Castration

**DOI:** 10.1007/s10508-022-02434-0

**Published:** 2022-11-03

**Authors:** Erik Wibowo, Samantha T. S. Wong, Richard J. Wassersug, Thomas W. Johnson

**Affiliations:** 1 Department of Anatomy, University of Otago, 270 Great King St., Dunedin, 9016 New Zealand; 2 Department of Educational and Counselling Psychology, McGill University, Montreal, PQ Canada; 3 Department of Cellular and Physiological Sciences, University of British Columbia, Vancouver, BC Canada; 4 Department of Anthropology (Emeritus), California State University, Chico, CA USA

**Keywords:** Genital ablation, Castration, Eunuchs, Body integrity dysphoria, Gender dysphoria

## Abstract

Some men elect castration voluntarily without any clear medical reason. Here we aim to document their perception of genital ablation and injuries to better understand their motivations for castration. Participants completed an online survey with open-ended questions related to their perspectives on castration, genital ablation, and genital injuries. Thematic analyses were performed on the responses to these questions. Responses were obtained from 208 male castrated individuals (51.9 ± 16.0 years old). Among these, 154 were physically castrated, 36 chemically castrated, and 18 nullified (had testicles and penis removed). The majority learned about castration from media (55.8%) or animal castration (23.4%). The circumstances when they first wanted to be castrated varied greatly. Most (46.3%) wished to achieve an idealized self motivated by gender dysphoria, body integrity dysphoria, or wanting to be conspicuously non-sexual. The top themes we identified related to the respondents’ perceptions of the pros of genital ablation were physical appearance, psychological benefit (i.e., a “eunuch calm”), and being non-sexual. Conversely, themes related to the cons they saw in having no genitals ranged from no disadvantages to loss of sexual/reproductive capability. Some perceived performing genital injury as a step toward ultimate castration or nullification. The respondents similarly varied in whether they saw any loss in having non-functional testicles. Perceptions in this regard appeared to differ depending on whether the respondents were taking supplemental androgens post-castration. Motivations for castration vary greatly between individuals. Clinicians need to understand men’s diverse perceptions on castration in order to provide appropriate care for individuals with strong castration desire.

## Introduction

Although uncommon, there are men with a strong desire to have their genitals removed with neither any clear medical need nor a diagnosis of male-to-female gender dysphoria (Johnson & Irwig, 2014; Wong et al., 2021). The desire is typically for an orchiectomy, but may include a desire for a penectomy as well (Brett et al., 2007; Franke & Rush, 2007; Mellon et al., 1989; Stunell et al., 2006; Volkmer & Maier, 2002; Wassersug & Johnson, 2007). Our team has previously surveyed individuals who wish to be castrated but did not desire feminization (Brett et al., 2007; Handy et al., 2016; Johnson et al., 2007; Vale et al., 2013). Many of these individuals wish to be eunuchs and privately identify as such, although they continue to present publicly as men. In that regard, they are unlike eunuchs in history, who were typically castrated before puberty and thus lacked most male secondary sexual characteristics (Johnson & Wassersug, 2010; Wassersug et al., 2012).

The motivations for voluntary genital ablation vary. Many modern-day voluntary eunuchs desire androgen suppression to lower their libido (Wassersug et al., 2004). This has been identified as male-to-eunuch (MtE) gender dysphoria (Vale et al., 2010; Wassersug et al., 2004). Men with such dysphoria wish to remove post-pubescent masculine features that are testosterone-dependent such as erections and sexual desire. However, they do not have any desire to be feminized. Some express a desire to revert to a prepubertal penis size with no desire for female genitalia. Some, however, appear to have Body Integrity Dysphoria (BID) defined as “…intense feelings of inappropriateness concerning current non-disabled body configuration” (6C21) in the International Classification of Diseases-11 (World Health Organization, 2019). In those instances, the individuals consider their external genitalia to be aberrant appendages that should be removed. Other motivations include religion-based celibacy, and to fulfil a sexual fantasy; i.e., they envision this as the ultimate sacrifice for a sexual submissive individual (Johnson & Irwig, 2014; Piccolo et al., 2019, 2022; Wong et al., 2021). In sum, there may be more than one reason that an adult male may desire genital ablation in the absence of any reproductive or genital pathology.

In terms of the etiology, some aspects of early childhood experience may contribute to the desire for genital ablation. These past experiences include ones of both a traumatic and non-traumatic nature. Vale et al. (2013) found that there is a higher likelihood for males who desire genital ablation to have had a religious upbringing or experienced childhood. Other childhood experiences include being threatened with castration by an adult, and/or having witnessed animal castrations. Furthermore, for some, the frequency of playing with male action figures that lack genitals (e.g., Ken doll, GI Joe) during childhood may have contributed to their castration desire (Wong et al., 2021).

Furthermore, genital injuries directed at the penis, scrotum, or testicles, some of which were self-performed, are not uncommon among individuals with castration desire (Jackowich et al., 2014). In some cases these injuries require hospitalizations (Johnson & Irwig, 2014). Anecdotally, some members on the Eunuch Archive—a website for people interested in castration-related topics—have noted that they attempted such injuries to gain access to a proper surgical orchiectomy, when they were unable to find a surgeon who are willing to perform the procedure, or when medical insurance would not cover the procedure without a valid medical reason.

Here, we conducted exploratory research to better understand what genital injuries and ablation mean to men who have been voluntarily castrated. Specifically, we performed thematic analyses on responses to open-ended questions on these topics in an anonymous survey for men who claimed to be voluntarily castrated. Their answers enrich our previous findings on factors that influence men’s desire for genital ablation (Vale et al., 2013; Wong et al., 2021). Most previous studies in this population used quantitative approaches, but in the current paper, we take a qualitative approach to capture more in-depth information on the participants’ perceptions on genital injury and ablation.

We specifically focus on what individuals, who had obtained voluntary genital ablation or injury, believed were the pros and cons of genital injury and ablation. Our goal is to better understand the various reasons why some men seek castration without any medical reasons and go as far as to injure their own genitals. Their answers provide insights to the men’s understanding of the potential risks of genital ablation or injuries.

Many men, who have had their testes removed, subsequently go on supplemental hormones to mitigate the adverse effects of hypogonadism. These hormones can be either estrogens or androgens. The most appropriate hormonal treatments may vary based on the dominant reason that the men sought castration. That, in turn, may be influenced by the men’s perception of the costs and benefits of castration. Thus we examine, not just the overall perception the men have of the pros and cons of castration, but how those perceptions align with the supplemental hormones that they may have elected to take post-castration.

The information provided here has direct implication to providing appropriate healthcare to men who have elected emasculating surgery outside a standard diagnosis of male-to-female gender dysphoria.

## Method

### Participants and Procedure

We surveyed members of the Eunuch Archive Internet community. Data were collected between October 2016 and June 2017. The Institutional Review Board of California State University—Chico, and the Eunuch Archive Steering Committee approved the research. The survey was built on the SurveyMonkey platform and an invitation with the survey’s URL was posted on online.

The invitation to take the survey mentioned: “The survey is for eunuchs, eunuch wannabes [i.e., intact males who are considering castration], and any others who may have interests in castration, whether as fantasy or academic interest.” After consenting, participants viewed the full survey, which took 30–40 min to complete. Participants did not receive any compensation for joining the study.

One thousand and twenty-three individuals completed the questionnaire. Wong et al. (2021) has described the exclusion for the data analyses. For this paper, we analysed feedback from 208 individuals who reported to have been castrated. We did not analyze responses from the remaining participants; i.e., those without castration desire, those who only had sexual fantasies about castration, and those who wanted to be castrated but had not pursued the procedure. The rationale for this exclusion is a practical one; i.e., the analyses would greatly lengthen the paper with information tangential to the core questions we wished to address. Data from these other groups would need to be analyzed separately.

## Measures

### Demographics

We included standard demographic questions to acquire data on age, ethnicity, relationship status, marital status, education, income, gender, country of residence, partner’s age, partner’s gender, religion, current and childhood living conditions, and sexual attraction based on the Kinsey Scale (1948). Options for sexual attraction were “X-Asexual or nonsexual (no interest in sexual activity with others)”, “0-Exclusively heterosexual with no homosexual attraction”, “1-Predominantly heterosexual, only incidentally homosexual”, “2-Predominantly heterosexual, but more than incidentally homosexual”, “3-Bisexual (Equal heterosexual and homosexual attraction)”, “4-Predominantly homosexual, but more than incidentally heterosexual”, “5-Predominantly homosexual, only incidentally heterosexual”, and “6-Exclusively homosexual”.

In addition, we also asked their castration status: “What is your current castration status or interest in castration?”. Four of the relevant answer options were “Surgically castrated: both testicles have been removed and are no longer present”, “Physically castrated: both testicles have been destroyed, but remnants are still present. Use of burdizzo [a large clamp for castrating farm animals by crushing the spermatic cord], injections of alcohol, etc. to destroy them”, “Nullified: Both penis and testicles have been removed”, and “Chemically castrated: Currently using ‘reversible’ chemical castrating drugs such as Lupron, Androcur, estrogen, etc.” Other responses are not shown here because they were for those who were not castrated, and data from those participants were removed in this paper. The numbers of those surgically and physically castrated in the survey are combined, because both are permanently castrated rather than treated reversibly by chemical means.”

#### Open-Ended Questions

We asked the following questions (also listed in Table 2): “How did you first learn about castration?”, “What were the circumstances when you first thought that you might want to be castrated?”, “What do you believe are the advantages of not having genitals?”, “What do you believe are the disadvantages of not having genitals?”, “What do you believe are the advantages of injuring genitals?”, “What do you believe are the disadvantages of injuring genitals?”, “What do you believe are the advantages of having nonfunctional testicles?”, and “What do you believe are the disadvantages of having nonfunctional testicles?”. We added the two last questions, unrelated to surgery, because some voluntary eunuchs have reported injecting toxins into their testes. They did this to kill testicular tissue as a prelude to getting a diagnosis of testicular pathology that warranted surgical intervention and a subsequent orchiectomy (Johnson & Irwig, 2014).

### Data Analysis

Demographic data are summarized using descriptive statistics. The answers to the open-ended questions were analysed using the thematic analyses framework outlined in Braun and Clarke (2006) with themes coded at the semantic level. The analysis was done by reporting the experiences as described by the participants. Initial responses were read and coded by EW. Response from one person can include multiple themes. Those themes and their proportion in relation to the total number of responses for each question are summarized in Table 2. Each theme is described in this paper, and each extract includes the participant number (noted with a (P…)). Words corrected for spelling errors and explanation for abbreviations are indicated within square brackets. Responses which were reported by fewer than 5% of participants were listed on Table 2 (compiled under “Other” category) but are not described in the Results.

## Results

### Demographics

Table 1 summarizes the demographic data of the participants. Participants were physically (74.0%), chemically (17.3%) castrated, or nullified (both testicles and penis surgically removed (- 8.7%).

**Table 1:** Demographic characteristics of voluntarily castrated individuals (*n* = 208)

	No ART	On ART	Total
Castration method			
Physically castrated	80 (62.5%)	74 (92.5%)	154 (74.0%)
Chemically castrated	35 (27.3%)	1 (1.3%)	36 (17.3%)
Nullified (Both testicles and penis removed)	13 (10.2%)	5 (6.3%)	18 (8.7%)
Mean age (± SD)	50.2 ± 17.3	54.8 ± 13.2	51.9 ± 16.0
Age of castration	46.1 ± 17.2	48.5 ± 12.3	47.3 ± 14.9
Self-identified gender (%)			
Male	38 (29.7%)	36 (45.0%)	74 (35.6%)
Eunuch	49 (38.3%)	36 (45.0%)	85 (40.9%)
Male-to-Female Transsexual	21 (16.4%)	1 (1.3%)	22 (10.6%)
Female	7 (5.5%)	0	7 (3.4%)
Genderqueer	6 (4.7%)	2 (2.5%)	8 (3.8%)
Others (e.g. genderfluid, androgynous, etc.)	7 (5.5%)	4 (5.0%)	11 (5.3%)
Missing	0	1 (1.3%)	1 (0.5%)
Ethnicity (%)			
White	114 (89.1%)	76 (95.0%)	190 (91.3%)
East Asian	6 (4.7%)	2 (2.5%)	8 (3.8%)
Others	7 (5.5%)	2 (2.5%)	9 (4.3%)
Missing	1 (.8%)	0	1 (0.5%)
In a relationship	76 (59.4%)	54 (67.5%)	130 (62.5%)
Marital status (%)			
Never married	47 (36.7%)	17 (31.5%)	64 (30.8%)
Married or civil union	57 (44.5%)	43 (53.8%)	100 (48.1%)
Separated	6 (4.7%)	3 (5.6%)	9 (4.3%)
Divorced	14 (10.9%)	11 (20.4%)	25 (12.0%)
Widowed	4 (3.1%)	4 (7.4%)	8 (3.8%)
Missing	0	2 (2.5%)	2 (1.0%)
Partner’s gender for those in a relationship (%)			
Female	62 (81.6%)	43 (79.6%)	105 (80.8%)
Male	8 (10.5%)	11 (20.4%)	19 (14.6%)
Others (e.g. eunuch, genderqueer, etc.)	5 (6.6%)	0	5 (3.8%)
Missing	1 (1.3%)	0	1 (0.8%)
Country of residence (%)			
USA	70 (54.7%)	47 (58.8%)	117 (56.3%)
United Kingdom	10 (7.8%)	9 (11.3%)	19 (9.1%)
Germany	8 (6.3%)	4 (5.0%)	12 (5.8%)
Canada	6 (4.7%)	6 (7.5%)	12 (5.8%)
Australia	7 (5.5%)	3 (3.8%)	10 (4.8%)
Others	25 (19.5%)	11 (13.8%)	36 (17.3%)
Missing	2 (1.6%)	0	2 (1.0%)
Childhood living condition			
Non-rural	99 (78%)	50 (62.5%)	149 (71.6%)
Rural	28 (22%)	30 (37.5%)	58 (27.9%)
Missing	1 (0.8%)	0	1 (0.5%)
Current living condition			
Non-rural	100 (79%)	58 (72.5%)	158 (76.0%)
Rural	27 (21%)	21 (26.3%)	48 (23.1%)
Missing	1 (0.8%)	1 (1.3%)	2 (1.0%)
Highest education attainment (%)			
High school degree	15 (11.7%)	12 (15.0%)	27 (13.0%)
Training from vocational/trade/business school	16 (12.5%)	9 (11.3%)	25 (12.0%)
College degree	38 (29.7%)	12 (15.0%)	50 (24.0%)
University degree			
Bachelor’s degree	22 (17.2%)	21 (26.3%)	43 (20.7%)
Master’s degree	25 (19.5%)	16 (20.0%)	41 (19.7%)
Doctoral degree	12 (9.4%)	10 (12.5%)	22 (10.6%)
Income (%)			
Less than $10,000	16 (12.5%)	1 (1.3%)	17 (8.2%)
Between $10,001–$30,000	28 (21.9%)	10 (12.5%)	38 (18.3%)
Between $30,001–60,000	34 (26.6%)	29 (36.3%)	63 (30.3%)
Between $60,001–100,000	29 (22.7%)	20 (25.0%)	49 (23.6%)
More than $100,000	20 (15.6%)	18 (22.5%)	38 (18.3%)
Missing	1 (0.8%)	2 (2.5%)	3 (1.4%)
Kinsey scale for sexual attraction			
0	20 (15.6%)	13 (16.3%)	33 (15.9%)
1	17 (13.3%)	13 (16.3%)	30 (14.4%)
2	9 (7.0%)	5 (6.3%)	14 (6.7%)
3	17 (13.3%)	12 (15.0%)	29 (13.9%)
4	7 (5.5%)	6 (7.5%)	13 (6.3%)
5	8 (6.3%)	9 (11.3%)	17 (8.2%)
6	16 (12.5%)	12 (15.0%)	28 (13.5%
X	33 (25.8%)	10 (12.5%)	43 (20.7%)
Missing	1 (0.8%)	0	1 (0.5%)
Religion			
Christian	56 (44%)	43 (53.8%)	99 (47.6%)
Other religion	12 (9%)	5 (6.3%)	17 (8.2%)
Non-religious	60 (47%)	32 (40.0%)	92 (44.2%)

*ART* = Androgen replacement therapy

Their mean age of castration was 47.3 ± 14.9 years old, which was close to the mean of their current age (51.9 ± 16.0). The majority identified as either male (35.6%) or eunuch (40.9%). The majority (91.3%) were White. Although less than half (48.1%) were married or in a civil union, 62.5% of the participants were in a relationship and primarily with a female (80.8%). The most common country of origin was USA (56.3%). Furthermore, 27.9% spent their childhood in a rural location and 23.1% remained in a rural place. A large proportion of the participants had a university education (51.0%) and had a household income of over $60,000 (41.9%).

In terms of their sexual attraction, 15.9% had exclusive attraction to females, 13.5% had exclusive attraction to males, and 20.7% were asexual. The proportion of asexual individuals was higher among those not on androgens (25.8%) than among those on androgens (12.5%). Of note, 13.9% claimed equal level of attraction to both males and females. Lastly, 47.6% were Christian, 8.2% followed other religions, and 44.2% were non-religious.

### Open-Ended Questions

Summary themes from the thematic analyses from the open-ended questions are listed on Table 2.

**Table 2:** Summary themes for responses to open-ended questions from men who received castration voluntarily

Themes for responses to each question	No ART	On ART	Overall
*How did you first learn about castration?*	(122 responses)	(75 responses)	(197 responses)
Media (e.g., TV, book, magazine, erotica, bible, researching the topic)	70 (57.4%)	40 (53.30%)	110 (55.8%)
Animal castration/Living in a farm	26 (21.3%)	20 (26.7%)	46 (23.4%)
Interaction with someone (e.g., discussion with someone, learning in school, threatened or injured by someone, doctor)	20 (16.4%)	10 (13.3%)	30 (15.2%)
Other (e.g., don’t remember, being a transgender, being castrated)	13 (10.7%)	6 (8.0%)	19 (9.6%)
*What were the circumstances when you first thought that you might want to be castrated?*	(113 responses)	(75 responses)	(188 responses)
To achieve a preferred self (e.g., gender dysphoria, body dysmorphia, to be non-sexual)	59 (52.2%)	29 (38.7%)	87 (46.3%)
Interaction with another person (e.g., partner is encouraging it, discussing castration with someone, relationship issues)	21 (18.6%)*	5 (6.7%)	26 (13.8%)
Eroticizing castration (e.g., sexual fantasy, BDSM practice, to be a better bottom)	19 (16.8%)	6 (8.0%)	25 (13.3%)
Media (e.g., reading contents in book, magazine, internet)	13 (11.5%)	8 (10.7%)	21 (11.2%)
Health reasons (e.g., testicular torsion, testicular pain, prostate cancer)	1 (0.9%)***	13 (17.3%)	14 (7.4%)
Other (e.g., don’t remember, thinking/seeing of girls peeing)	13 (11.5%)	16 (21.3%)	29 (15.4%)
*What do you believe are the advantages of not having genitals?*	(111 responses)	(73 responses)	(184 responses)
Physical appearance (e.g., genital region appears smooth, genital region appears better)	39 (35.1%)	30 (41.1%)	69 (35.1%)
Psychological (e.g., feeling free, calm, happy, align with gender, being sexual in other way)	35 (31.5%)	17 (23.3%)	52 (28.3%)
Become non-sexual (e.g., to reduce thinking about sex and masturbation)	30 (27.0%)	16 (21.9%)	46 (25.0%)
Medical-related benefits (e.g., lower prostate cancer risk, ‘lower risk for STI’, infertile, absent of T effects)	15 (13.5%)	13 (17.8%)	28 (15.2%)
Other (e.g., nothing, not sure, no advantage)	8 (7.2%)	8 (11.0%)	16 (8.7%)
*What do you believe are the disadvantages of not having genitals?*	(104 responses)	(69 responses)	(173 responses)
No disadvantages	39 (37.5%)	18 (26.1%)	57 (32.9%)
Sexual/reproductive dysfunction (e.g., weaker orgasm, lower sexual desire, infertility)	25 (24.0%)	20 (29.0%)	45 (26.0%)
Social (e.g., being ‘odd’, perceived as not normal, difficulty in relationships)	16 (15.4%)	7 (10.1%)	23 (13.3%)
Side effects related to androgen loss (e.g., weight gain, osteoporosis, fatigue)	13 (12.5%)	7 (10.1%)	20 (11.6%)
Urinating problems (e.g., having to sit down or to squat while urinating)	9 (8.7%)	4 (5.8%)	9 (8.7%)
Other (e.g., don’t know, frustration, reporting benefit)	12 (11.5%)	17 (24.6%)	29 (16.8%)
*What do you believe are the advantages of injuring genitals?*	(102 responses)	(68 responses)	(170 responses)
No advantage	44 (43.1%)	24 (35.3%)	68 (40.0%)
As a way to have genital ablation	30 (29.4%)*	30 (44.1%)	60 (35.3%)
Experiencing the effects of androgens deprivation (lower testosterone levels, lower sexual function)	14 (13.7%)	6 (8.8%)	20 (11.8%)
Sexual pleasure (e.g., sexual excitation, erotic pain)	4 (3.9%)	7 (10.3%)	11 (6.5%)
Other (e.g., aligning with gender)	11 (10.8%)	6 (8.8%)	17 (10.0%)
*What do you believe are the disadvantages of injuring genitals?*	(98 responses)	(66 responses)	(164 responses)
Health risks (e.g., blood, pain, infection, infertility, loss of sensation)	62 (63.3%)	43 (65.2%)	105 (64.0%)
No disadvantages	18 (18.4%)	13 (19.7%)	31 (18.9%)
Social (e.g., embarrassment, having to explain to people)	8 (8.2%)	5 (7.6%)	13 (7.9%)
Other (e.g., don’t know, just want it removed)	10 (10.2%)	9 (13.6%)	19 (11.6%)
*What do you believe are the advantages of having nonfunctional testicles?*	(108 responses)	(70 responses)	(178 responses)
Achieving genital ablation and its effect (e.g., testosterone loss, smooth genital area, infertility)	31 (28.7%)	27 (38.6%)	58 (32.6%)
Becoming non-sexual (e.g., low libido, no erections)	36 (33.3%)*	13 (18.6%)	49 (27.5%)
Psychosocial benefit (e.g., calm, not aggressive, happy, sexual pleasure with partner)	36 (33.3%)***	6 (8.6%)	42 (23.6%)
No benefit at all	11 (10.2%)**	20 (28.6%)	31 (17.4%)
Other (e.g., not sure, not applicable)	7 (6.5%)	7 (10.0%)	14 (7.9%)
*What do you believe are the disadvantages of having nonfunctional testicles?*	(97 responses)	(67 responses)	(164 responses)
No disadvantages	37 (38.1%)	21 (31.3%)	58 (35.4%)
Side effects related to androgen loss (e.g., weight gain, osteoporosis, fatigue)	29 (29.9%)	24 (35.8%)	53 (32.3%)
Sexual and reproduction effects (e.g., loss of erection, libido, ability to have children)	18 (18.6%)	18 (26.9%)	36 (22.0%)
Physical appearance (e.g., the testes are still present)	5 (5.2%)	4 (6.0%)	9 (5.5%)
Other (e.g., don’t know, answers were advantages rather than disadvantages)	11 (11.3%)	12 (17.9%)	23 (14.0%)

Questions are presented in italics

Themes in “Other” categories were reported by < 5% of the respondents. The percentages do not add up to 100% because some responses fit multiple themes

*ART* = androgen replacement therapy

*Significantly different proportion of responses with a particular theme than in those who received ART, *P* < .05; **P < .01; ***P < .001

### Question: How Did You First Learn About Castration?

Participants first learned about castration in a variety of ways (197 responses). The most commonly reported theme (55.8%) was through the media, for example through “…a horror film” (P27), “I read about the sex change Christine Jorgensen had in Denmark which included castration” (P280), and “BME [Body Modification E-zine]” (P353). Among these responses with “media” theme, 13 out of 110 responses had erotic elements, such as “reading through erotic fantasy stories” (P718), and “I actually was watching porn cartoon” (P160). Only 2 out of 110 reported having learned about castration from the Bible.

The second most reported introduction to the idea of castration came from witnessing animal castration or from living on a farm (23.4%), such as “saw farmer castrate cows” (P261) and “castrating farm animals” (P245). The third most common theme is from interacting with someone (15.2%). These included non-specified discussions as well as being threatened with castration by someone, in a meeting with doctor, or learning about it in school. Example quotes include “aunt threatened for undone zipper” (P108), “a conversation in a bar” (P991), and “a friend was sharing a story he read about a young man who was castrated for pay” (P860).

### Question: What Were the Circumstances When You First Thought You Might Want to Be Castrated?

For this question, 188 participants provided responses. The primary theme is to achieve their preferred self (46.3%). Among these, there were three sub-themes: gender dysphoria (25.3%), BID (37.9%), or to be non-sexual (20.7%). Example quotes include “seemed right for me, I do not like having genitals” (P726), “gender dysphoria during school years” (P26), and “I wanted to get away from all of the sexual feelings that I was having all the time” (P32).

Additional themes which were reported by 7–14% of the respondents included:

1. Interaction with another person (e.g., “my wife …encouraged my castration fantasy… She kept encouraging it, as a way to control my sex drive” (P36), and “our washing machine broke had no clean underwear and my mom made me wear my sisters panties for a few days” (P477)).
2. Eroticization of castration (e.g., “sexually excited reading [BME] and eunuch archive” (P253), and “I discovered as a gay man that I was exclusively a bottom sexually, and my testicles were a distraction to full enjoyment to gay sex” (P997)).
3. From accessing the media including books and the Internet (e.g., “reading on the Internet (BME & other) I found the idea very interesting” (P83), and “going through eunuch.org I learnt about castration and got motivated” (P39)).
4. Health reasons that were treated with surgical castration such as testicular torsion, prostate cancer, and acute testicular pain.

### Having No Genitals

In this study, 184 participants provided feedback on what they perceived as the advantages in having no genitals. Three main themes reported by 25–35% of the respondents were:

1. Obtaining a physical benefit such as smooth appearance, no possibility for testicular injuries, or simply not physically having the genitals (e.g., “female clothing fits better, lack of anything to injure by blow to crotch…” (P517), and “smooth profile; clothes fit better; safer (no chance of getting "kicked in the nuts"); more comfortable; better appearance” (P508)).
2. Psychological benefits such as being less aggressive, aligning with their gender better, or being able to be sexual in a different way (e.g., “I feel my true self identity” (P997), “calm, less aggressive” (P289), and “unique and very erotic inducing to others” (P135)).
3. To be non-sexual, including to reduce sexual desire and sexual activities (e.g., “reducing the focus of sex to get a normal life” (P572), and “…I don't waste so much time, thinking about sex, masturbating, and watching porn” (P954)).

In addition, 15.2% perceived some medical benefits (e.g., “less chance of cancer” (P814), and “…almost zero risk of STD [sexually transmitted diseases]” (P872)).

Participants also provided feedback on what they believed as disadvantages for lacking genitals (173 responses). A large proportion (32.9%) did not feel there is any disadvantage in not having genitals, such as “cannot think of any” (P45), “none” (P586), and “nothing” (P979). Themes reported by 8–26% of participants were:

1. Sexual or reproductive problems (e.g., “orgasm is mostly less or down. It’s very difficult to get an erection. The erection is mostly too short to penetrate a female. Your [sexual] drive for all things is less or down…” (P572), and “sterility” (P187).
2. Social concerns such as being perceived as unusual or relationship difficulty (e.g., “hiding from others, meaning unable to use public locker room” (P346), and “no longer can please your partner in that way” (P640).
3. Side effects related to androgen loss (e.g., “physically weaker, lack endurance. Hot flashes” (P954), and “missing hormones might lead to several diseases (osteoporosis, less strength, depression, weight gain etc.)…” (P123)).
4. Issues with urination (e.g., “not being able to piss properly” (P1), and “[difficulty] urinating without squatting” (P97)).

### Genital Injury

In our sample, 170 participants provided opinions on what they perceived as advantages of injuring genitals. A large proportion (40.0%) did not perceive any advantage of injuring genitals, such as “none” (P954), “nothing” (P495), and “no advantages” (P860). Three main themes for the remaining responses were:

1. As a way to obtain genital ablation (e.g., “to be a fast way of ridding myself of these ugly things” (P195), “none, except that the injury [allowed] me to be surgically castrated [safely] in hospital” (P353), and “it is a means to have surgical castration done with health insurance paying the cost, rather than paying out-of-pocket” (P997)).
2. Effects associated with androgen deprivation (e.g., “to destroy their ability to produce testosterone and sperm” (P814), “lack of erection” (P173), and “no sex drive” (P160).
3. Sexual pleasure associated with inciting pain on genitals (e.g., “erotic pain” (P143), “…pleasure pain” (863), and “Very erotic in nature, also filthy indeed” (P135).

We also obtained data from 164 participants on what they perceived as disadvantages of injuring genitals. The main theme of the responses was related to health risks associated with genital injuries such as pain, infection, infertility, and bleeding (64.0%). Example quotes included “it hurts” (P187), “no hormones” (P495), and “…risk of infection or other complications” (P866).

Additionally, 18.9% of responses perceived no disadvantages of injuring genitals (e.g., “none” (P339), “nothing” (P979), and “for myself, none” (P918)). Furthermore, 7.9% provided responses related to social impact of having no genitals (e.g., “embarrassing doctor visits” (P108), and “explaining injuries” (P764)).

### Having Non-Functional Testicles

We received 178 responses on the advantages of having non-functional testicles. The top three themes were achieving of genital ablation and its effects (32.6%), to become non-sexual (27.5%), psychosocial benefits (23.6%).

A few examples for the first theme included “no unwanted children. No excess sex drive” (P108), “no T production. Nice smooth flat look” (P27), and “no testosterone… tucking is easier since [I] now don't have to shove my testicles into my inguinal canal. also, no possible children. i don't want children.” (P29). Examples for the non-sexual theme included “total relief from his sex drive. No more [hard-ons]” (P149), and “not be led by [libido] and being in a calm state” (P927). In addition, some quotes for the psychological benefits included “less "macho" feelings; more relaxed generally” (P868), “calm, less aggressive” (P289), “reducing dysphoria” (P946), and “being free to focus on your sex partner's pleasure” (P749).

Furthermore, 17.4% did not perceive any benefit in having non-functional testicles, such as “there are no benefits in itself” (P997), and “none, they should not be there causing damage” (P661).

For the feedback on disadvantages of having non-functional testicles, we obtained 164 responses. A large proportion (35.4%) perceived no disadvantages of having non-functional testicles, such as “none, maybe I can get them removed” (P631), and “so far, none” (P866). Other reported themes included:

1. Side effects associated with androgen deprivation (e.g., “depression maybe? osteoporosis” (P14), “[initially] hot flashes gynaecomastia” (P11), and “weight issues, loss of strength, [osteoporosis]” (P100))
2. Sexual and reproductive problems (e.g., “no cum” (P640), “lack of ability to perform sexually (when you want to perform in that capacity)” (P508), “no more can make a child” (P555), and “unable to procreate” (P973))
3. Physical appearance of still having testicles (e.g., “they're still hanging there” (P517), “…you want but can't get them removed (that's my case) it's really disgusting” (P872), and “bulge” (P427))

### Comparison of Themes Based on Participants’ Use of Androgen Replacement Therapy

In this study, 80 out of 208 participants were on androgen replacement therapy (ART), and we noted a few differences in their responses to those without ART. As noted on Table 1, 92.5% participants on ART were physically castrated as compared to 62.5% among those without ART (*χ*^2^(1) = 23.1, *p* < 0.001). Concerning the circumstances when participants first wanted to be castrated, there were significant differences in proportions of responses with themes “interaction with another person” (18.6% without ART vs. 6.7% on ART; *χ*^2^(1) = 5.4, *p* < 0.05) and “health reason” (0.9% without ART vs. 17.3% on ART; *χ*^2^(1) = 17.7, *p* < 0.001). Furthermore, there were proportionally more androgen-deprived participants (15.9%) reporting gender dysphoria in response to this question than those on ART (5.3%; *χ*^2^(1) = 4.9, *p* < 0.05), suggesting that those on ART are more likely to have BID.

For the advantages of injuring genitals, participants without ART (29.4%) reported proportionally fewer responses with the theme “as a way to have genital ablation” (*χ*^2^(1) = 3.9, *p*  < 0.05) than those on ART (44.1%).

Lastly, regarding the perceived advantages of having nonfunctional testicles, there were significant difference in the proportions for the themes “becoming non-sexual” (33.3% without ART vs. 18.6% on ART; *χ*^2^(1) = 4.6, *p* < 0.05), “psychological benefit” (33.3% without ART vs. 8.6% on ART; *χ*^2^(1) = 14.4, *p* < 0.001), and “no benefit at all” (10.2% without ART vs. 28.6% on ART; *χ*^2^(1) = 10.0, *p* < 0.01).

## Discussion

There are several main findings from this study. The majority of voluntary eunuchs learned about castration either from the media or from first-hand experience with animal castration. The situations when they first had castration desire varied greatly, with the primary theme for wanting castration to achieve a preferred self, be it motivated by gender dysphoria, BID or a desire to be non-sexual.

Despite knowing the disadvantages of not having genitals, many voluntary eunuchs perceived various advantages of lacking genitals that included a smooth genital appearance, improved psychological well-being, and becoming non-sexual. In addition, while a large proportion did not believe that injuring their genitals (without removing them) has any benefits, some believed that injuring their genitals opened a pathway to eventually achieve a medically safe genital ablation. Conversely, while some wanted to injure their genitals to have lower sexual function, some found sexual pleasure in injuring their genitals. The majority of the voluntary eunuchs in our study seemed aware of the potential risks associated with genital injury, such as pain, infection, and sexual dysfunction.

Most voluntary eunuchs nevertheless perceived benefits of having non-functioning testicles, such as becoming non-sexual, and improving psychological well-being. Again, although many perceived no disadvantages of having non-functioning testicles, the majority were aware of possible disadvantages, such as the various physiological adverse effects of hypogonadism. In sum, our findings show that voluntary eunuchs generally are aware of the risk for genital ablations, but despite this, also perceive that genital ablation would benefit them in various ways.

### Learning About Castration

The most common pathway for learning about castration was either via media or knowledge of animal castration. The media examples included movies, history books, the Bible, and various websites. For example, some respondents mentioned reading about eunuchs in Chinese history or watching documentaries about transgender people. Furthermore, a large proportion of voluntary eunuchs were raised in a devout Christian household (Vale et al., 2013), so they may have come across castration in the Bible. In the current study, 44% identified as Christian but only two reported first learning of castration from the Bible. There are also some (13 out of 110) who became aware of castration from reading erotic stories or watching erotic materials with a sadomasochistic castration theme.

The second dominant theme—of learning castration from animal castration—was reported by 23.4% participants. This response is consistent with the fact that 27.9% grew up in a rural setting where farm animal castration is a common practice. As an aside, this proportion is approximately two times the proportion of US residents who live in rural areas (Dobis et al., 2021).

### Gender Dysphoria or Body Integrity Dysphoria

The situation when participants recall first desiring castration varies greatly, emphasizing that the etiology for castration desire is complex and diverse. Gender dysphoria, BID, and a desire to be non-sexual have been previously described in the literature (Johnson & Irwig, 2014) and are seen in the quotes from participants in our study. Most of our participants may be categorized as either having MtE gender dysphoria or BID. The former are more likely to be the ones not on ART subsequent to their castration, as they wish to be free from the effects of testosterone. Evidence to support such categorization comes from the difference in proportion for certain themes. For example, proportionally, more participants not on ART thought their circumstance for wanting to be castrated had gender dysphoria than those on ART.

In addition, more participants without ART perceived that nonfunctional testicles benefit them by making them non-sexual and by giving them psychological benefits. In the Eunuch Archive community, this is often called a “eunuch calm” which has been loosely defined as having both reduced libido and less reactive aggression (Wassersug et al., 2004). This result supports the idea that their goal for genital ablation is to reduce the effects of androgens in their body, instead of to remove their genitals per se. Indeed, some participants perceived the benefit of not having genitals is to better align with their gender and to become non-sexual, which is consistent with MtE gender dysphoria (Johnson & Wassersug, 2010; Vale et al., 2010).

In contrast, the motivation to be castrated for participants on ART is more consistent with BID (“…intense feelings of inappropriateness concerning current non-disabled body configuration”) (World Health Organization, 2019). This is supported by the finding that 44.1% of participants who went on ART subsequent to their castration perceived genital injury as helping them to eventually obtain proper and safe genital ablation. In contrast, only 29.4% of those who did not go on ART perceived such benefit from genital injury. These findings strengthen the notion that many of those on ART wish to physically remove their genitals due to BID, but not necessarily lower their sexual drive or function.

Furthermore, many participants also considered that not having genitals improved their physical appearance—i.e., to have smooth genital appearance that lacks testicles. This description fits a BID diagnosis (Johnson & Irwig, 2014; Vale et al., 2010). This population may feel that their genitals do not belong to their body and wish to have them removed.

As mentioned in our previous study, there may be some whose desire to being castrated was influenced by having played with male action figures without genitals (Wong et al., 2021). This reappears in the current data, but the number of respondents reporting this is few (< 5%).

We also noted that a few participants on ART learned about castration because of a medical reason. There is a possibility that they may have neither MtE gender dysphoria nor BID, but that their castration was due to medical necessity or perceived medical issue, such as testicular pain.

### Genital Injuries

More than half of our participants were aware of the risk of injuring their genitals and is noteworthy that ~ 35% perceived that injuring their genitals would eventually lead to genital ablation. Indeed we know that some voluntary eunuchs injured their genitals so they could get proper medical treatment and care (Johnson & Irwig, 2014). As also mentioned in the Methods, we included questions on the pros and cons of non-functional testicles, because some voluntary eunuchs made their testicles non-functional. A common way to do this is to inject toxins (Johnson & Irwig, 2014) as a gambit for eventually getting medical intervention leading to a proper orchiectomy. Some participants in our sample envisioned that having injured testicles would be an effective step toward obtaining more complete genital ablation in a proper medical setting.

Participants also were aware of the different consequences for not having genitals or having non-functional testicles, although ~ 35% perceived no disadvantage of not having genitals or having non-functional testicles. Despite knowing of disadvantages, our participants had achieved genital ablations. Currently, we do not know if they knew these disadvantages *before* having genital ablation, or if they became aware of some of these *after* having genital ablation. Suffice to say, our population had a castration desire strong enough for them to pursue genital ablation including self-surgery if proper medical care was not available.

It is noteworthy that the standard of care document (SoC) version 7 from the World Professional Association for Transgender Health (WPATH) offered no recommendation for clinicians on how to care for someone with a strong desire for genital ablation desire yet no desire for feminization. Vale et al. (2010) has previously highlighted the importance of having such a guideline, especially since many people with such desires undergo ablative procedures by unqualified persons, either as surgery upon themselves or underground ‘cutters’ (Jackowich et al., 2014; Johnson & Irwig, 2014). The new WPATH SoC version 8, however, provides a new dedicated chapter for this population. That chapter includes recommendations for health professionals to offer medical and surgical intervention when there is a high risk that withholding treatment would lead to harm such as through self-injury, surgery by unqualified person, or unsupervised hormonal use (Coleman et al., 2022).

### Castration Paraphilia

As noted above, an additional reason for wishing to be castrated appears to be as sexual fantasy that eroticizes castration (Piccolo et al., 2019, 2022). Others have similarly documented that extreme desires for ablative surgeries may be grounded in sexualizing the body modification (Blom et al., 2017). The origin of such a fetish may vary, such as from castration-themed erotica, having castration paraphilia, or being encouraged by their sexual partner. The data that we collected in this survey are the first to show a link between the sexualized themes in the EA stories and real life motivation for castration among individuals who have themselves been voluntarily castrated.

Whereas a small number of participants reported having first learned about castration from erotica (13 participants; 6.6%), twice of that proportion reported that their desire for genital ablation emerged from an eroticization of castration (25 participants; 13.3%). However, these data need to be taken with caution. It is possible that the actual proportions were higher because those with castration desire are more likely to be sexually attracted to castrated men and feel attractive without testicles (Wibowo et al., 2022). Thus, they may have sought castration-related erotica.

Indeed, as also noted by Handy et al. (2016), some voluntary eunuchs have sexual attraction to and fantasies about castrated men. Given that there are people with erotic target inversion (Brown et al., 2020), it is likely that some of the voluntary eunuchs are aroused by the idea or fantasy of being castrated in accord with that common theme in EA stories (Piccolo et al., 2022). Our recent data (Wibowo et al., 2022) indicated that individuals with desire for genital ablation (be it castration or penectomy) are also at higher odds for being attracted to men without genitals. For a few participants, their castration desire was induced by their sexual partner encouraging it. It remains to be determined how such an interaction would lead to someone having an extreme desire for genital ablation.

The eroticization of castration can be an extreme paraphilia, which may indeed lead some individuals to eventually get themselves castrated. At the same time, individuals may seek castration to get control of that same paraphilia when it leads to repeated genital self-injury and is overwhelming their lives.

### Limitations

Our study has several limitations. First, since data were collected online, we could not validate the accuracy or biases in some responses (e.g., whether or not they were castrated, or if those on chemical castrating drugs were on a dose sufficient enough to induce androgen deprivation). An example of these potential bias includes when participants did not remember accurately details from their youth or childhood. They might also have some reluctance or embarrassment with disclosing sexual information. In addition, some individuals might not fully recognize, or accept any sexual nature to their castration desire. Other responses which may be sensitive to recall bias include information about how respondents first learned about castration and circumstances that raised their desire for castration. Here, we asked for participants’ age at the start of the survey and year of birth at the end to reduce the risk of unreliable data (i.e., if one answered the questions consistently). We removed data from individuals with discrepancies in those data. Since we recruited participants from a website dedicated to castration topics, our data may only be generalizable to individuals with elevated interest in genital ablation. In addition, only one of us coded the data, thus we could not indicate inter-rater reliability. Lastly, the open-ended questions analyzed here were not administered by an interviewer, but rather through an online survey. Having them asked by an interviewer would allow us to gain more insights, and clarify responses that lead to further questions.

### Conclusion

The motivations for voluntary eunuchs to seek castration varies between individuals. However, despite knowing the disadvantages of injuring their genitals or lacking testicles, they also perceive advantages. For them, the perceived advantages of castration outweighed the disadvantages. Clinicians need to be aware that there are men with strong castration desire who will find a way to fulfil their wish of castration. Unfortunately, there is not yet any SoC for men who are or desire to be voluntary eunuchs. Clinicians may not know how to provide appropriate endocrine or surgical treatment for them. The upcoming SoC document from the WPATH should in part address this, and it hopefully will better serve such populations.

Clinicians need to consider how to best provide care to clients desiring castration. As noted above, the client’s motivation for genital ablation may depend on the reason for their desire. Men with MtE gender dysphoria may simply wish to be free of the psychoneuroendocrinological effects of testosterone. In contrast, those with BID may have a strong desire to physically remove their testicles because they feel their testicles are not an appropriate part of their body. They may nevertheless wish to retain the psychological effects of testosterone, such as normal libido. As such, those with accurate BID diagnosis may be best managed with ART subsequent to their castration.

In meeting the needs of men with either a history of genital self-injury or a desire for testicular ablation, clinicians need to be aware of the diversity of motivation for genital ablation. Also, to meet the healthcare needs of such men so that they can make informed decisions on both their surgery and hormonal management, clinicians need to understand the diversity of factors contributing to the desire for genital injury and ablation.

## Data Availability

Data are not archived in a repository.
